# Supplementary material for: Calcium promotes persistent soil organic matter by altering microbial transformation of plant litter
Source: Nat Commun. 2023 Oct 19;14:6609. doi: 10.1038/s41467-023-42291-6 (PMC10587086; doi:10.1038/s41467-023-42291-6)
Supplement: Supplementary file 1 — Supplementary Information [file 41467_2023_42291_MOESM1_ESM.pdf]

Supplementary Information

Calcium promotes persistent soil organic matter by altering microbial transformation of plant litter

Itamar A. Shabtai[1,2,*,#], Roland C. Wilhelm[1,3], Steffen A. Schweizer[4], Carmen Höschen[4], Daniel H. Buckley[1,5], and Johannes Lehmann[1,6]

*Corresponding author

[1]Soil and Crop Sciences, School of Integrative Plant Science, Cornell University, Ithaca, NY, 14850, USA

[2]Department of Environmental Science and Forestry, The Connecticut Agricultural Experiment Station, New Haven, CT, 0651

[3]Department of Agronomy, College of Agriculture, Purdue University, West Lafayette IN 47907, USA

[4]Chair of Soil Science, School of Life Sciences, Technical University of Munich, 85354, Freising, Germany

[5]Department of Microbiology, Cornell University, Ithaca NY USA

[6]Cornell Atkinson Center for Sustainability, Cornell University, Ithaca, NY, 14850, USA

[#] Current address: Department of Environmental Sciences, The Connecticut Agricultural Experiment Station, New Haven, CT, 0651

Supplementary methods

Heterotrophic respiration measurement

The amount of CO_2 produced in each microcosm was determined using the KOH method. Qorpak vials containing the samples were placed in 473 mL Mason jars along with 20 mL scintillation vials which contained a CO_2 trap (15 mL KOH, 0.18 M) made with pre-boiled, CO_2-free deionized water. CO_2-free deionized water (5 mL) was added to the bottom of each Mason jar to maintain a humid atmosphere. Measurements from blank jars with no soil (n = 3) were used to account for the small amount of CO_2 present in the jar. On measurement days, the jars were opened, and the electrical conductivity of the KOH solutions was determined. After each measurement, the CO_2 traps were replaced with fresh KOH solutions in new vials. At each sampling event, the mean electrical conductivity value of the KOH solution from the blank jars was subtracted from each jar's KOH electrical conductivity value. This corrected value was then converted into CO_2 volume using a calibration curve, and further converted to mass by applying the ideal gas law. A calibration curve was calculated for each batch of KOH solution by injecting known volumes of CO_2 (99.99%) into Mason jars which had septa installed in their lids containing empty Qorpak vials and KOH traps. The electrical conductivity of the KOH solution was measured 24 hours after injection.

To measure the $\delta^{13}C$ values of the emitted CO_2, the KOH solutions were transferred (after their electrical conductivity was measured) to 50 mL conical vials containing 5 mL of 0.3 M $BaCl_2$, resulting in $Ba^{13}CO_3$ precipitation. The vials were centrifuged at 2500 rpm for 5 min, and the supernatant was decanted. The precipitate was rinsed with CO_2-free deionized water (10 mL), centrifuged again, and the supernatant was decanted, for a total of three rinses. The remaining precipitate was dried at 70 °C. The entirety of precipitated $Ba^{13}CO_3$ from days 1, 2, 3, and 4 was

composited as one single sample for each jar, and the precipitated $Ba^{13}CO_3$ from days 6, 8, 11, 15, 21, 30, 44, 61, 91, 112, (and 135 for 40% WFPS samples) was composited as another single sample for each jar. The composited $Ba^{13}CO_3$ samples therefore provide an integrated measurement of the $\delta^{13}CO_2$ respired from each jar at T_{beg} (days 0-4) and T_{end} (days 5-end). $Ba^{13}CO_3$ samples were acidified using H_3PO_4 and the released CO_2 was analyzed for $\delta^{13}C$ on a Thermo Scientific DELTA V isotope ratio mass spectrometer interfaced with a Gasbench II (Thermo Scientific, West Palm Beach, FL).

C K-edge and Ca L-edge NEXAFS

Samples were prepared by pressing pellets into the SGM Beamline sample holders. No binder was used in the process. Carbon Partial Fluorescence Yield (PFY) spectra were collected using a silicon drift detector (Amptek FastSDD) placed in the plane of polarization at 90 degrees to the incident beam to minimize the contribution of scattering in the measurement. Multiple (between 10 and 30) 60 second long slew scans were acquired on different spots on the samples and averaged to obtain the raw PFY measurement. Normalization of the raw PFY is critical for the C K-edge measurements due to the energy dependence in the transmission of the beamline arising from carbon contamination of the beamline optics. To accurately measure the I_0 (incident flux) at the C K-edge, a measurement of the Boron fluorescence from highly pure Boron Nitride is used. The intensity of B partial fluorescence yield at the C K-edge closely follows the intensity of the incident beam, aside from a slight decrease in the B attenuation cross section between 270 and 320 eV. The normalized C PFY is produced by dividing the raw PFY by this B PFY. Energy calibration of the spectra was checked using the C1s (C=O) to pi* transition at 288.7 eV of a citric acid standard. The spectra were baseline corrected by fitting a linear regression through the pre-edge region (270-275 eV) in Fityk ver. 1.3.1[1], and subtracting the intercept from the spectra

to achieve a near-zero pre edge. Next, step-edge normalization was done in Athena ver. 0.9.26[2] by fitting a linear line through the pre edge (270-280 eV) and a second-order polynomial through the post-edge (303-320 eV), using 289.2 eV as E0. SOC composition was assessed by deconvoluting the spectra (280-305 eV) using a Gaussian function fitting algorithm in Fityk ver. 1.3.1[1]. An arctangent function was used to model the ionization step at 290 eV with full width at half maximum (FWHM) set at 0.5 eV, and eight Gaussian peaks (FWHM 0.6 eV, floated amplitude), constituting C functional groups[3]. Additionally, two σ^* transitions at 290.2 eV and 291.5 eV were simulated using Gaussian curves. Peak heights of modeled C NEXAFS spectra were used to evaluate differences among samples.

The Ca L-edge spectra were energy corrected using the Ca 2p transition of a calcite ($CaCO_3$) standard at 352.6 eV, baseline corrected as above, and normalized using the in-line I_o. Edge-step normalization was done in Athena ver. 0.9.26[2] using a pre-edge range of 340.6-345.6 eV, post-edge range of 354.6 - 360 eV, E0 of 352.6 eV, and a third-order polynomial. Ca L-edge NEXAFS spectroscopy of the soil samples showed two major peaks (349.4 and 352.6 eV) that are characteristic of spin-orbital partners found in Ca L-edge NEXAFS, and two smaller peaks preceding the larger peaks at 348.2 and 351.4 eV, which correspond to the crystallinity of the Ca structures (Supplementary Fig. 2). A splitting ratio, defined at the ratio of absorbance intensity of each smaller peak (348.2 and 351.4 eV) and the intensity of the minimum separating this peak from the corresponding main peak (349.4 and 352.6 eV), was used to quantify crystallinity (Supplementary Fig. 3)[4]. Based on the position of the two major peaks, and the splitting ratio our results show that the spectra of Ca-treated and control soils were similar at T_{beg} and T_{end}, and in soils to which litter was added, or not. Compared to reference standards (Supplementary Fig. 4), our samples highly visually resembled Ca-smectite and organic-Ca salts, and were most

dissimilar to the spectrum of $CaCl_2$, which had a peak at 349.2 eV and high intensity at 348.2 and 351.4 eV, evidence of its highly crystalline structure. Thes findings indicate that soil Ca was non-crystalline and was likely interacting with phyllosilicates and organics. We did not detect changes in the Ca L-edge following Ca addition.

Infrared spectromicroscopy

Microaggregates (53 – 250 µm) were collected by dry sieving, water saturated by misting with ultrapure water, and frozen at -20 °C[5]. Multiple sections (500 nm) were cut from each microaggregate at -55°C using an ultramicrotome (Leica EM UC7, equipped with a Leica EM FC7 cryostage) with a diamond knife (Cryo 45 °C, Diatome). Sections were transferred to IR grade CaF_2 circular windows (25 mm diameter, 1 mm thick, Crystran, UK) using a Perfect Loop (Electron Microscopy Sciences, US), and air-dried. Spectra were acquired in transmission mode using a 128 X 128-pixel focal plane array detector equipped with a mercury cadmium telluride detector, with 25X magnification, and an effective pixel size of 3.3 µm. Spectra were obtained by coadding 128 scans collected from 3900 to 900 cm^{-1}, at a spectral resolution of 4 cm^{-1}. Spectral data were processed using Quasar[6]. ROIs (4-5) were manually selected and only spectra with > 0.4 absorbance units at 1035 cm^{-1} were kept, representing well defined mineral sections. Selected spectra from each ROI were then baseline corrected using the Rubberband method and scattering corrected using the EMSC function (polynomial order 2, no scaling) with the mean spectrum as reference. To establish equal sample size for each linear model, 2000 observations were randomly sampled from each sample.

NanoSIMS

***Sample preparation*.** Samples were air-dried, impregnated with an acetone-resin mixture, and then embedded in epoxy resin (Araldite 502) according to Herrmann et al.[7]. Thin sections were

cut and polished to obtain a flat sample surface. The sections were coated with approximately 10 nm Au/Pd under Ar atmosphere before being analyzed by scanning electron microscopy (SEM) to select representative regions of interest at the interface of litter fragments and fine-textured regions similarly in all treatments (Supplementary Fig. 14A). Before NanoSIMS analysis, the sections were coated with approximately 30 nm Au/Pd under Ar atmosphere.

Image analysis

First, we corrected the raw data for electron multiplier dead-time using the OpenMIMS plugin in FIJI[8,9]. In addition to the attempted matching of the optics of the two primary ion sources, a transformation of the Cs measurements was done to match the O data. For this, the BigWarp plugin was used to create a landmark image using $^{16}O^{-}$ and $^{27}Al^{+}$ image (or $^{44}Ca^{16}O^{-}$ and $^{44}Ca^{+}$). The affine transformation algorithm was transferred to all other ion channels based on the linear transformation with translation, rotation, independent scales, and shearing[10].

To quantify the co-localization of various spatial units of the imaged soil structures, supervised segmentations were conducted based on a machine-learning algorithm in Ilastik 1.2[11]. The segmentation considered various image features including the intensity, texture, and local gradient in all respective ion channels [12]. The O measurements were segmented using the $^{56}Fe^{+}$, $^{40}Ca^{+}$+$^{44}Ca^{+}$, $^{27}Al^{+}$ distributions to differentiate Ca, Fe, and Al segments as well as regions that were not related to any of those three elements (termed ‘none’ in Figure 6 and Supplementary Fig. 14E). The segmentation of the warped Cs measurements was done using $^{16}O^{-}$, $^{12}C^{-}$+$^{13}C^{-}$, and $^{12}C^{14}N^{-}$+$^{12}C^{15}N^{-}$ to differentiate mineral-dominated regions (high O), OM-dominated regions (high C and CN), and the resin matrix impregnating the pores (high C and low CN) (Supplementary Fig. 8E). The segmentation of ^{15}N-enriched spots was done independently using the ^{15}N enrichment ratio ($^{12}C^{15}N^{-}$ / ($^{12}C^{14}N^{-}$+$^{12}C^{15}N^{-}$) with a contrast enhanced between 0-3%.

Based on the high Ca counts at OM-dominated regions, only the mineral-dominated regions were separately segmented into Ca, Fe, Al segments based on the O measurements. By combining both segmentations, the co-localization of mineral-dominated and OM-dominated regions with Ca, Fe, and Al segments was quantifiable (Supplementary Fig. 8F). The Si:(Al+Si) ratio of the 'none' segments in the mineral-regions was approximately five times higher than the Ca, Al, or Fe regions, indicating that they are quartz rich particles, while in the OM-dominated regions, there was no specific enrichment of any of the metals in the 'none' regions, indicating OM-rich segments. We calculated the relative pixel proportions of spatial co-localization types (excluding the 'none' segments which did not correlate with any metal) and the ion ratios to compare their composition (Figure 6D-H and Supplementary Fig. 14). The total area of soil sections imaged with both primary ion sources was 0.23 mm^2.

DNA extraction and amplicon sequencing

DNA was extracted from soil using the 'DNeasy PowerSoil HTP 96 Kit', as per the manufacturer's instructions (QIAGEN, Germantown, MD) with a bead beating treatment of 3 min at 5.5 m s^{-1} (Bio Spec Products, Santa Clara, CA). Soil DNA extracts were quantified using the Quant-iT™ PicoGreen™ dsDNA Assay Kit (Thermo Fisher Scientific, Inc., Waltham, MA) and measured with a FilterMax F5 micro-plate reader (Molecular Devices, San Jose, CA). Bacterial community composition was determined by sequencing the V4 region of the 16S rRNA gene amplified from soil DNA extracts by polymerase chain reaction (PCR) using dual-indexed barcoded 515f/806r primers[13]. PCR was performed with 2 ng of DNA template, in triplicate, according to methods previously described[14] and pooled, purified and normalized to a standard concentration using the SequalPrep normalization kit (Invitrogen, CA, USA). Amplicon libraries were multiplexed and sequenced on a single lane of Illumina MiSeq (2 × 250 paired-

end) with a spike-in of 8% PhiX at the Biotechnology Resource Centre (Cornell University, Ithaca, NY, USA).

Additional characterizations of changes in bacterial community structure

DNA yield

After four days of incubation, DNA yields were significantly higher in soils amended with litter; generally lower where calcium was added; and did not differ according to soil moisture (Supplementary Fig. 12). The effect of calcium on DNA yield was more pronounced in soil with high moisture content (at T_{beg}).

Effects of treatment factors on alpha-diversity

Treatments had immediate and longer-term impacts on the richness and evenness of bacterial communities. At T_{beg}, the communities in Ca-treated soils amended had significantly lower species richness, yet this difference was no longer apparent at T_{end} (Supplementary Fig. 13A). At T_{beg}, Pielou's evenness was lowest where litter was amended to low moisture soils but did not significantly differ by Ca treatment. The evenness of all communities increased with time, while Ca-treated soils had slightly (and significantly) lower evenness (Supplementary Fig. 13B). Both species richness and evenness were significantly lower in litter amended soils at T_{beg}, indicating that the additional organic matter resource enriched for subpopulations of decomposers.

Effects of treatment factors on beta-diversity

Approximately a quarter of the total ASVs (23.4%; 852 / 3,635) were differentially abundant according to one or more treatments, representing about half of all reads ($\bar{x}$ = 53%; full list of differential abundant ASVs provided in a separate Supplementary Data file). The response

of the major bacterial groups favored by calcium was immediate and enduring, with the same taxonomic groups favored across time (Supplementary Fig. 10). Firmicutes populations favored by calcium were particularly enriched at the start of incubations where litter was added driven by populations of *Paenibacillus*, *Ammoniphilus*, *Aneurinibacillus*, unclassified Planococcaceae, and *Fictibacillus*. In general, ASVs that were differentially more abundant in litter amended soil were favored by high moisture content. Taxa that were favored by low moisture were more likely to also be favored by calcium amendment (Fisher's exact; O.R. = 1.9; p-value = 0.05). Taxa that were favored by litter amendment were somewhat more likely to be favored by calcium amendment, but this relationship was not significant (O.R. = 1.5; p-value = 0.16).

Evidence supporting role of surface colonizing bacteria

Several observations indicated that Ca treatment selected for populations adapted to surface colonization, though it is likely that a greater diversity of physiological traits underpin the observed shift in microbiome, including potential tolerance to salinity due to the transient influx of $CaCl_2$ prior to flushing. While phylogenetic gene marker data does not provide direct evidence of the physiological traits of bacteria, the taxonomic classification of surface-colonizing bacteria is generally reliable, since specialized growth habits for surface colonization (hyphae-formation), surface gliding motility, or dimorphic surface-adherent lifestyles are phylogenetically conserved[15,16]. Thus, we have confidence that the taxa favored by Ca possess traits of surface-colonization.

To gauge the potential role of salinity stress, we can assess the temporal response of bacterial groups, since any exposure to salinity stress during the pre-incubation was transient. We observed that most of the major surface-colonizing groups responding to Ca treatment (ex. *Hyphomicrobiales* and *Xanthomonadales*) had the same relative abundance in control and Ca-

treated soils at T_{beg} but diverged in Ca treated soils over time (Supplementary Fig. 11). This trend was captured by the increased amount of variation in community composition attributed to Ca treatment over time (Fig. 3). The one exception to the temporal trend in surface-colonizing populations was the hyphae-forming actinobacterial groups, which were most differentially enriched by Ca at T_{beg}, and less so after 4 months (Supplementary Fig. 11). These actinobacterial groups are recognized for their tolerance to osmotic stress, and it is plausible that exposure to high salinity during pre-incubation may have factored in their early high differential abundance. However, this does not preclude the importance of their growth habit. Hyphae-forming *Actinomycetota* are demonstrably favored in soils where pores are less hydraulically connected due to the competitive edge of hyphae-forming bacteria in traversing pore spaces in drier soils[17]. Importantly, the low moisture condition (40% WFPS) was not selected to represent a stress condition, but rather to fall within the lower range of non-drought conditions in soils. Thus, we consider the abundance patterns of *Actinomycetota* in our experiment as evidence of the advantage provided by their filamentous growth. Additional evidence includes:

1. The same *Actinomycetota* and surface-colonizing bacterial taxa enriched in Ca treated soils were among the major decomposers of particulate cellulose in a recent stable isotope probing experiment[18].
2. Calcium is a critical co-factor in many of the modes of attachment of bacteria favored by Ca in our study. For example, Ca is essential for the motility of surface-gliding bacteria, like *Sorangium* and *Halangium*[19,20]. Fibrobacterota degrade cellulose using enzymes that have enhanced substrate binding in the presence of calcium[21], with the addition of CaO to silage strongly selecting for Fibrobacterota in cow rumen[22]. Calcium also enhances the strength of adhesins and stimulates biofilm production[23,24] and is required for the optimal

growth of surface-adhering bacteria, including *Hyphomicrobium* [25]. Biofilms that form as part of surface colonies can also enhance water retention[26] and increase substrate diffusion into the microbial colony[27].

Tables and Figures

Supplementary Table 1. Properties of the field soil before and after preincubation treatments. Values are reported as mean(±sd) of three independent replicates.

Property (units)	Untreated soil	Ca-treated	Control soil
pH	6.1(±0.1)	6.04(±0.02)	6.24(±0.02)
EC (µS cm^{-1})	184(±19)	312(±7)	128(±16)
Ca (meq 100 g^{-1})	14.6(±0.15)	16.1(±0.15)	14.5(±0.2)
Mg (meq 100 g^{-1})	2.2(±0.07)	1.1(±0.05)	2.3(±0.05)
Na (meq 100 g^{-1})	0.1(±0)	0(±0)	0.1(±0)
K (meq 100 g^{-1})	0.2(±0)	0.2(±0)	0.2(±0)
CEC (meq 100 g^{-1})	17.1(±0.07)	17.4(±0.13)	17.1(±0.07)
$MWD_{slow-wet}$ (mm)	2.95(±0.01)	2.80(±0.02)	2.87(±0.01)
$MWD_{fast-wet}$ (mm)	2.32(±0.08)	1.94(±0.02)	1.95(±0.06)
Aggregate slaking value[a]	1.27(±0.08)	1.44(±0.03)	1.47(±0.09)
DOC (µg C g^{-1} soil)	n.d.	207(±9.5)	134(±6.9)
SOC (%)	3.81(±0.1)	3.83(±0.06)	3.78(±0.09)

[a] protocol according to Shabtai et al.[28]; higher values indicate lower aggregate stability

Supplementary Table 2. ANOVA model output and pairwise comparison between Control and Ca soil. C_{min} = mineralizability; ^{13}C-CO_2 = fraction of CO_2 from litter-C; ^{13}C-MBC = fraction of microbial biomass from litter-C; CUE = microbial carbon use efficiency; T_{beg} = Beginning of incubation (4 days); T_{end} = End of incubation (~4 months). P values indicating significant effect of Ca treatment are bolded. Pairwise comparisons are averaged across water content levels (Treatment x Water interaction not significant). Statistical significance was tested using unpaired, two-sided t-tests. Estimates (est.) have the same units as variable. Cohen's d effect size considered "large effect size" (>0.8) are bolded. Statistical tests were done on *n* =3 independent samples.

	**C_{min}**		**^{13}C-CO_2**		**^{13}C -MBC**		**CUE**	
**Fixed effect**	F	P	F	P	F	P	F	P
Treat	18.39	**0.0005**	0.067	0.79	19.004	**0.0005**	22.74	**0.0002**
Time	8166	<0.0001	103.49	<0.0001	52.848	<0.0001	285.27	<0.0001
Water	87.42	<0.0001	5.867	0.027	0.439	0.517	5.77	0.028
Treat x Time	0.59	0.45	1.058	0.32	3.664	0.07	15.73	0.001
Treat x Water	0.24	0.63	0.22	0.64	0.144	0.71	0.21	0.65
Time x Water	94.75	<0.0001	20.201	0.0003	1.588	0.23	0.0	0.98
Treat x Water x Time	0.02	0.89	2.694	0.12	0.607	0.45	2.012	0.17

Ca-control pairwise comparisons

	**C_{min}**			**^{13}C-CO_2**			**^{13}C -MBC**			**CUE**		
Time	P	est.	effect size	P	est.	effect size	P	est.	effect size	P	est.	effect size
T_{beg}	**0.039**	3.6	**1.41**	0.37	0.01	0.53	**0.01**	-0.16	**1.93**	**0.002**	-0.09	**2.58**
T_{end}	**0.006**	5.2	**1.05**	0.59	- 0.01	.031	**0.008**	-0.06	**2.04**	0.08	-0.01	**1.14**

Supplementary Table 3. ANOVA model output and pairwise comparison between Control and Ca soil. ^{13}C-MAOM = percent of mineral associated organic matter-C from litter-C; ^{15}N-MAOM = percent of MAOM-N from litter-N; ^{13}C:^{15}N-MAOM = MAOM C:N ratio from litter ^{13}C^{15}N; T_{beg} = Beginning of incubation (4 days); T_{end} = End of incubation (~4 months). P values indicating significant effect of Ca treatment are bolded. Pairwise comparisons are averaged across water content levels (Treatment x Water interaction not significant). Statistical significance was tested using unpaired, two-sided t-tests. Estimates (est.) have the same units as variable. Cohen's d effect size considered "large effect size" (>0.8) are bolded. Statistical tests were done on *n* =3 independent samples.

	**^{13}C-MAOM (%)**		**^{15}N-MAOM (%)**		**^{13}C:^{15}N-MAOM**	
**Fixed effect**	F	P	F	P	F	P
Treat	4.47	**0.050**	1.97	0.179	5.27	**0.036**
Time	76.46	<0.0001	195.5	<0.0001	257.96	<0.0001
Water	48.92	<0.0001	89.65	<0.0001	9.96	0.006
Treat x Time	1.49	0.24	1.45	0.24	4.23	0.056
Treat x Water	0.37	0.55	0.47	0.503	1.05	0.32
Time x Water	40.84	<0.0001	78.56	<0.0001	21.79	0.002
Treat x Water x Time	1.36	0.26	0.08	0.77	11.21	0.004

Ca-control pairwise comparisons

	**^{13}C-MAOM (%)**			**^{15}N-MAOM (%)**			**^{13}C:^{15}N-MAOM**		
Time	P	est.	effect size	P	est.	effect size	P	est.	effect size
T_{beg}	**0.014**	0.3	**1.81**	**0.043**	0.3	**1.39**	0.89	0.03	0.08
T_{end}	0.61	0.1	0.31	0.91	< 0.1	0.07	**0.0017**	0.52	**2.66**

Supplementary Table 4. ANOVA model output and pairwise comparison between Control and Ca soil. fPOM = amount of free particulate organic matter (mg g^{-1} soil); oPOM = amount of occluded particulate organic matter (mg g^{-1} soil); MAOM = amount of mineral associated organic matter (mg g^{-1} soil); T_{beg} = Beginning of incubation (4 days); T_{end} = End of incubation (~ 4 months). P values indicating significant effect of Ca treatment are bolded. Pairwise comparisons are averaged across water content levels (Treatment x Water interaction not significant). Statistical significance was tested using unpaired, two-sided t-tests. Estimates (est.) have the same units as variable. Cohen's d effect size considered "large effect size" (>0.8) are bolded. Statistical tests were done on *n* =6 independent samples.

	**fPOM (mg g^{-1} soil)**		**oPOM (mg g^{-1} soil)**		**MAOM (mg g^{-1} soil)**	
**Fixed effect**	F	P	F	P	F	P
Treat	0.79	0.38	4.18	**0.048**	0.56	0.46
Time	5.47	0.0246	42.63	<0.0001	3.78	0.06
Water	41.77	<0.0001	0.0	0.965	4.19	0.05
Treat x Time	0.12	0.73	0.16	0.69	2.78	0.10
Treat x Water	1.02	0.32	0.62	0.44	0.93	0.34
Time x Moisture	21.20	0.0001	13.12	0.0001	1.11	0.30
Treat x Water x Time	1.37	0.25	0.12	0.73	1.54	0.223

Control-Ca pairwise comparison

	**fPOM**			**oPOM**			**MAOM**		
Time	P	est.	effect size	P	est.	effect size	P	est.	effect size
T_{beg}	0.65	0.9	0.133	0.69	-0.5	0.07	0.26	10.9	0.34
T_{end}	0.99	0.01	0.001	**0.04**	1.8	0.35	0.21	-19.1	0.38

Supplementary Table 5. ANOVA model output and pairwise comparison between Control and Ca soil. ^{13}C-fPOM= fraction of fPOM-C from litter-C; ^{15}N-fPOM= fraction of fPOM-N from litter-N; ^{13}C-oPOM= fraction of oPOM-C from litter-C; ^{15}N-oPOM= percent of oPOM-N from litter-N; T_{beg} = Beginning of incubation (4 days); T_{end} = End of incubation (~4 months). P values indicating significant effect of Ca treatment are bolded. Pairwise comparisons are averaged across water content levels (Treatment x Water interaction not significant). Statistical significance was tested using unpaired, two-sided t-tests. Estimates (est.) have the same units as variable. Cohen's d effect size considered "large effect size" (>0.8) are bolded. Statistical tests were done on *n* =3 independent samples.

	**^{13}C-fPOM**		**^{15}N-fPOM**		**^{13}C-oPOM**		**^{15}N-oPOM**	
**Fixed effect**	F	P	F	P	F	P	F	P
Treat	0.04	0.84	0.0	0.99	5.88	**0.027**	6.33	**0.019**
Time	26.91	<0.0001	25.44	0.0001	5.13	0.038	3.2	0.09
Water	17.47	0.0007	25.02	0.0001	4.46	0.051	0.67	0.43
Treat x Time	0.71	0.41	1.64	0.21	1.73	0.207	0.46	0.51
Treat x Water	2.9	0.1	1.79	0.179	0.006	0.94	0.0	0.94
Time x Water	11.28	0.004	23.93	0.0001	19.78	0.0004	7.75	0.013
Treat x Water x Time	0.45	0.51	0.19	0.66	0.19	0.67	0.03	0.86

Control-Ca pairwise comparison

	**^{13}C-fPOM**			**^{15}N-fPOM**			**^{13}C-oPOM**			**^{15}N-oPOM**		
Time	P	est.	effect size	P	est.	effect size	P	est.	effect size	P	est.	effect size
T_{beg}	0.66	-0.02	0.25	0.23	-0.04	0.73	0.059	0.10	**1.23**	0.074	0.09	**1.15**
T_{end}	0.71	0.04	0.22	0.72	0.04	0.21	0.63	0.03	0.29	0.22	0.05	0.76

Supplementary Table 6. Peak heights of modeled C NEXAFS spectra from Ca-treated and control soils incubated with labeled litter at low or high water content, and sample at T_{beg} and T_{end}.

			Peak energy (eV)			
**Treatment**	**Time**	**Water content**	**285.3 aromatic**	**286.7 phenolic**	**288.7 carboxylic**	**289.4 O-alkyl**
Control	T_{beg}	low	0.323	0.481	1.013	0.848
Control	T_{beg}	high	0.327	0.491	1.015	0.859
Ca	T_{beg}	low	0.298	0.459	1.017	0.859
Ca	T_{beg}	high	0.316	0.508	1.022	0.854
Control	T_{end}	low	0.362	0.531	1.000	0.848
Control	T_{end}	high	0.341	0.530	1.000	0.850
Ca	T_{end}	low	0.354	0.548	0.999	0.831
Ca	T_{end}	high	0.351	0.544	0.999	0.829

Supplementary Table 7. Output of ANCOVA models comparing coefficients of linear regression models constructed from FTIR microscopy data for Ca-treated and control soils. The interaction coefficient size indicates the difference in slopes (control-Ca); a negative value indicates a smaller coefficient for a control soil. Also shown are the standard error and t values of the coefficient. Statistical significances, indicating that the slope differs significantly between groups, are tested using a one-sided F-test. Linear regressions were constructed from *n* = 2000 data points collected from at least 8 independent samples.

Functional group	Time	Interaction term	Estimated interaction coefficient	Std error of coefficient	t	P
Aromatic	T_{beg}	aromatic*treatment	-0.904	0.051	-17.84	< 0.0001
Carboxylic	T_{beg}	carboxylic *treatment	-0.139	0.017	-7.96	< 0.0001
Aliphatic	T_{beg}	aliphatic*treatment	-0.574	0.030	-18.94	< 0.0001
Aromatic	T_{end}	aromatic*treatment	-1.026	0.049	-21.12	< 0.0001
Carboxylic	T_{end}	carboxylic*treatment	-0.459	0.022	-21.22	< 0.0001
Aliphatic	T_{end}	aliphatic*treatment	-0.278	0.025	-11.27	< 0.0001

Supplementary Table 8. Output of ANCOVA models comparing coefficients of linear regression models constructed from FTIR microscopy data at T_{beg} and T_{end}. The interaction coefficient size indicates the difference in slopes (T_{end}-T_{beg}); a negative value indicates a smaller coefficient at T_{end}. Statistical significances, indicating that the slope differs significantly between groups, are tested using a one-sided F-test. Linear regressions were constructed from n = 2000 data points collected from at least 8 independent samples.

Functional group	Treatment	Interaction term	Estimated interaction coefficient	Std error of coefficient	t	P
Aromatic	Ca	aromatic*time	0.348	0.034	10.11	< 0.0001
Carboxylic	Ca	carboxylic *time	0.432	0.016	27.81	< 0.0001
Aliphatic	Ca	aliphatic*time	-0.091	0.023	-3.91	< 0.0001
Aromatic	Control	aromatic*time	0.226	0.061	3.71	0.00021
Carboxylic	Control	carboxylic*time	0.112	0.018	6.06	< 0.0001
Aliphatic	Control	aliphatic*time	0.205	0.025	8.35	< 0.0001

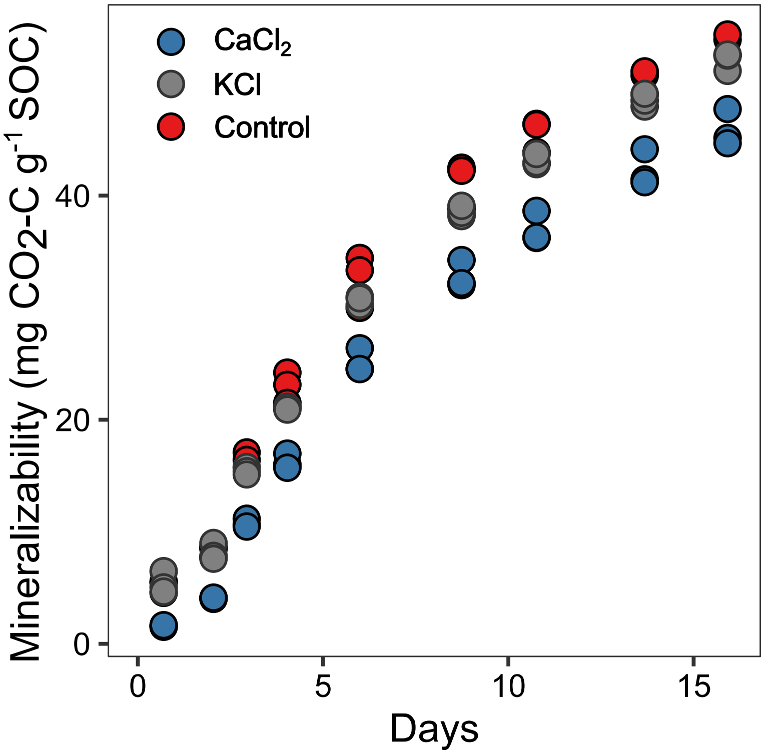

Supplementary Fig 1. Cumulative mineralizability of soils treated with $CaCl_2$ or KCl (200 meq L^{-1}), and a control soil, and incubated with plant litter (2% w/w) for 16 days. Three independent replicates are shown for each treatment.

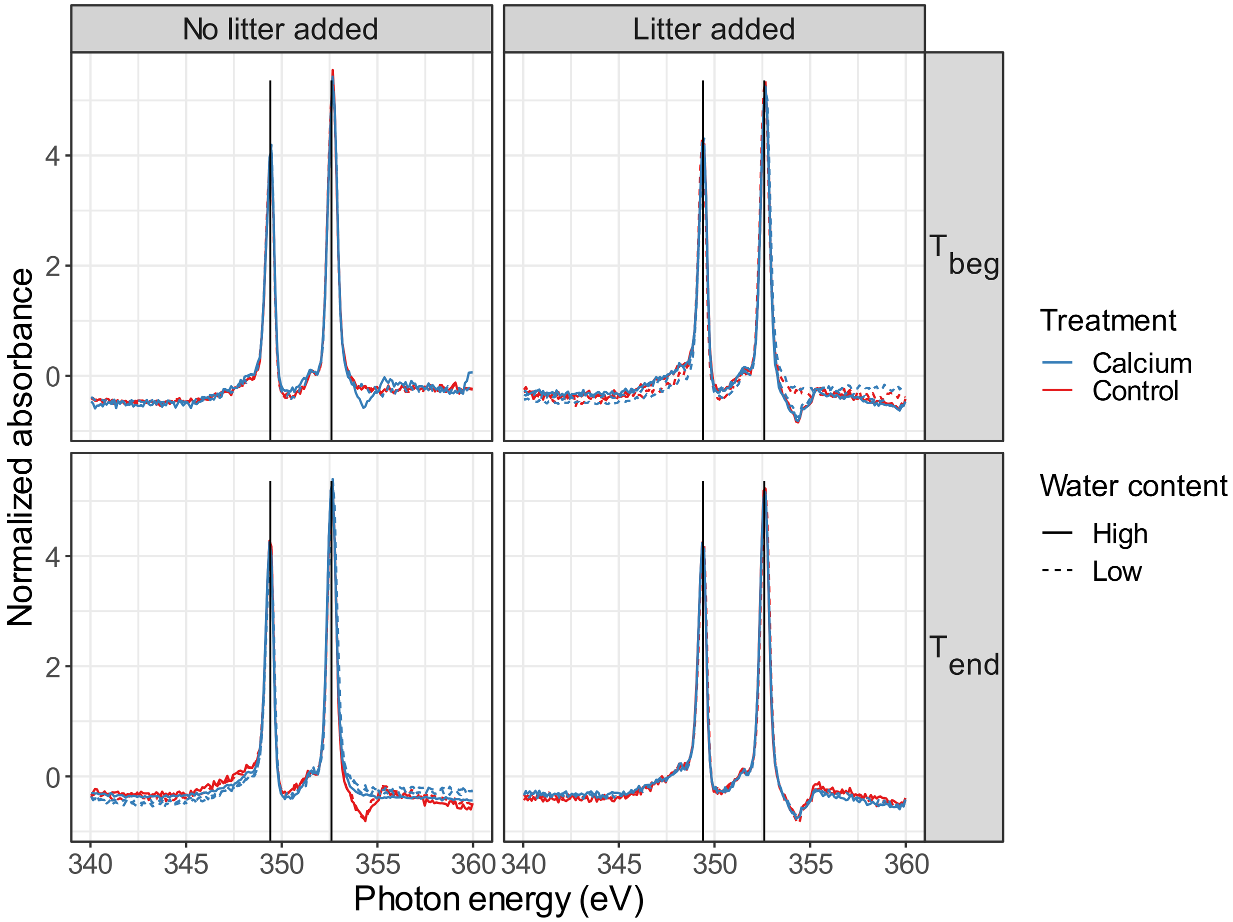

Supplementary Fig. 2. Ca L-edge NEXAFS spectra of Ca-treated and control soils incubated at low or high water content, sampled at T_{beg} and T_{end}.

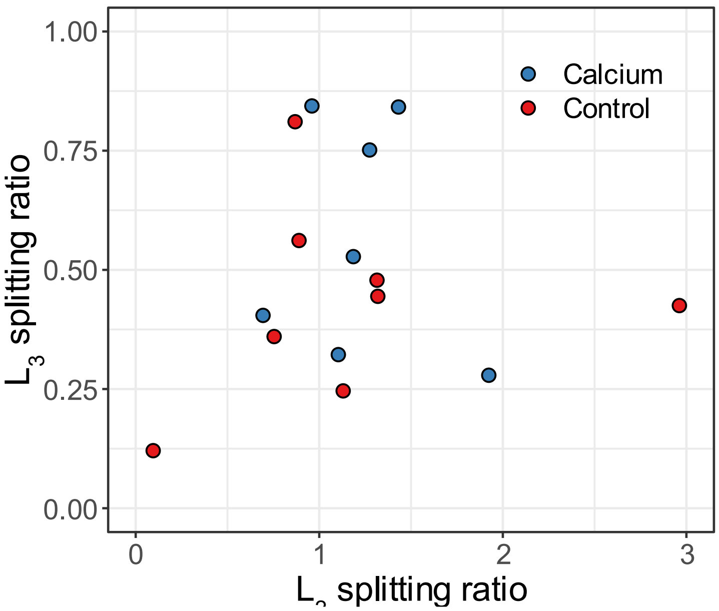

Supplementary Fig. 3. Scatterplot of L_2 vs L_3 splitting ratios in Ca-treated and control soils calculated from Ca L-edge NEXAFS. Lack of difference indicates similar crystallinity of Ca-structure, a chief characteristic of Ca L-edge NEXAFS.

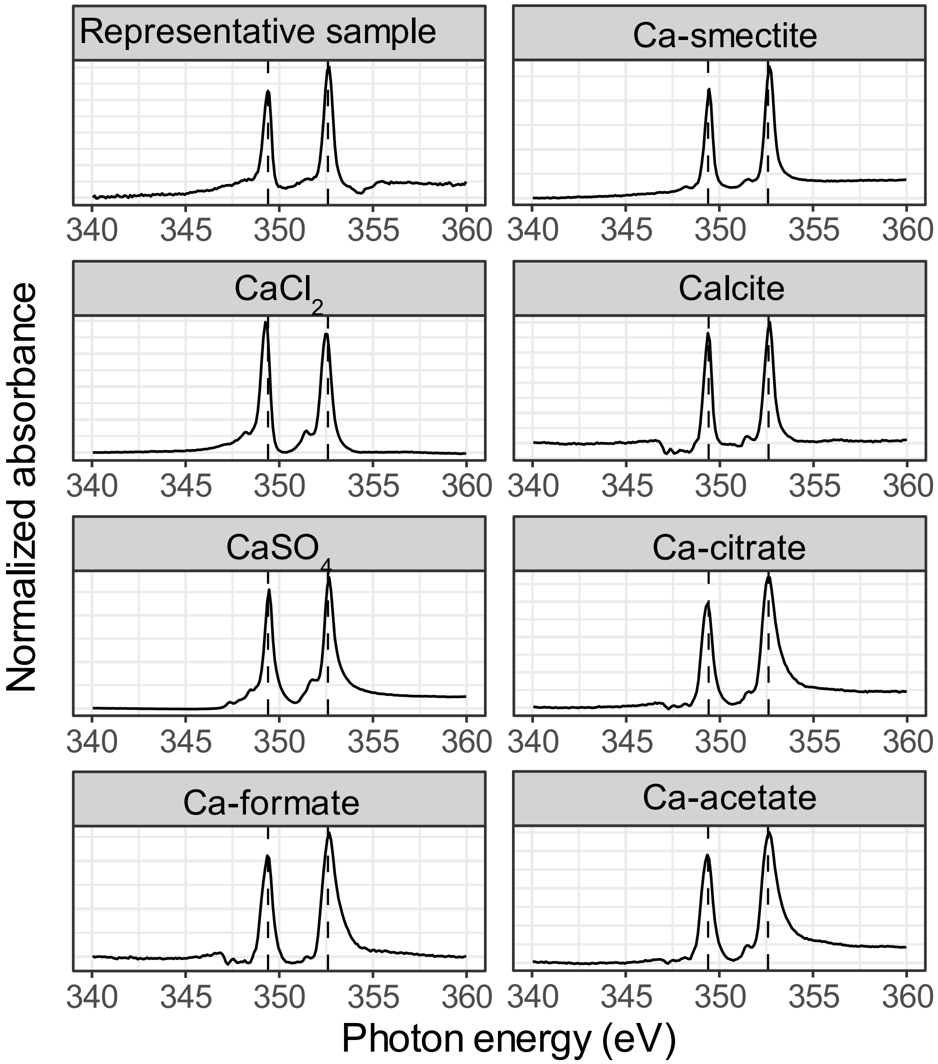

Supplementary Fig. 4. Ca L-edge NEXAFS spectra of a representative sample and reference Ca compounds. The representative sample was a Ca-treated soil sample, incubated with litter at low water content and sampled at T_{end}. Dashed vertical lines show the major peaks at 349.4 and 352.6 eV. Note the shift of the $CaCl_2$ spectrum from these peaks.

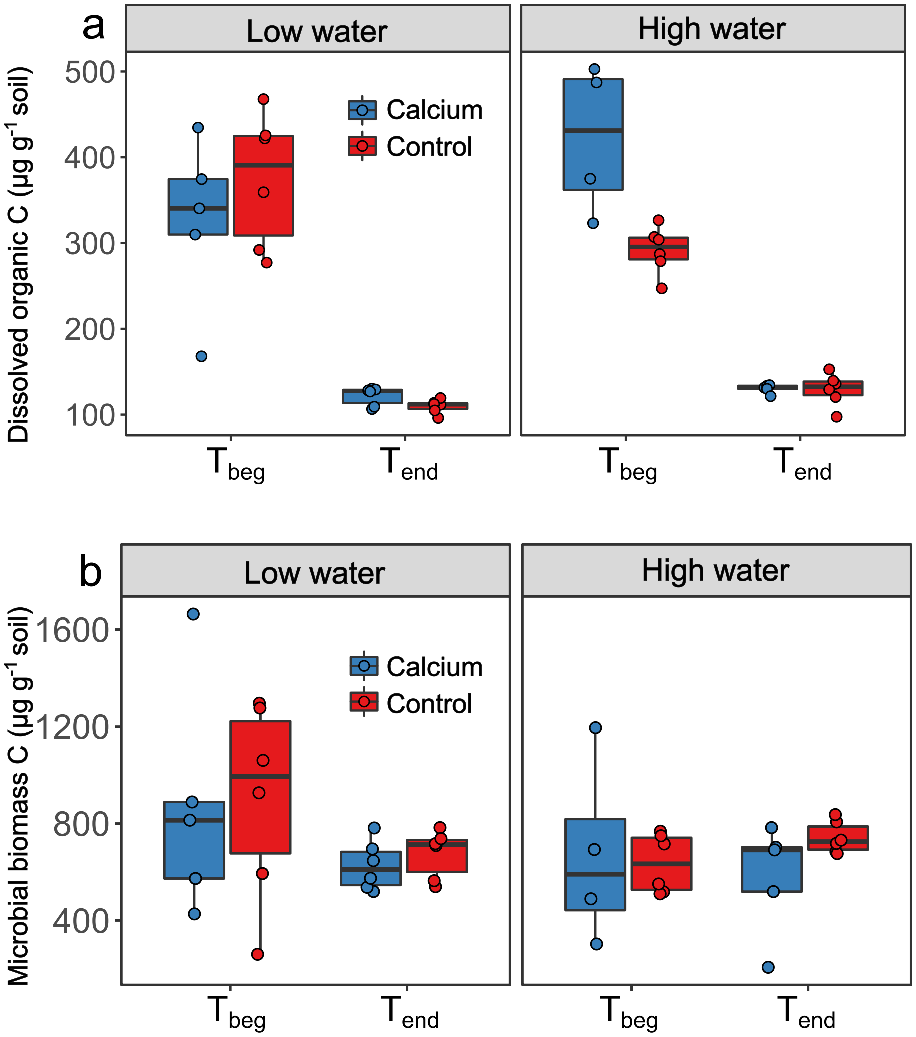

Supplementary Fig. 5. dissolved organic C (DOC) (a) and Microbial biomass C (MBC) (b) in Ca-treated and control soils, incubated with litter at low or high water content, and sampled at T_{beg} or T_{end}. No significant differences (*n* = 6 independent samples) were observed between Ca treated and control soils in any of the treatments.

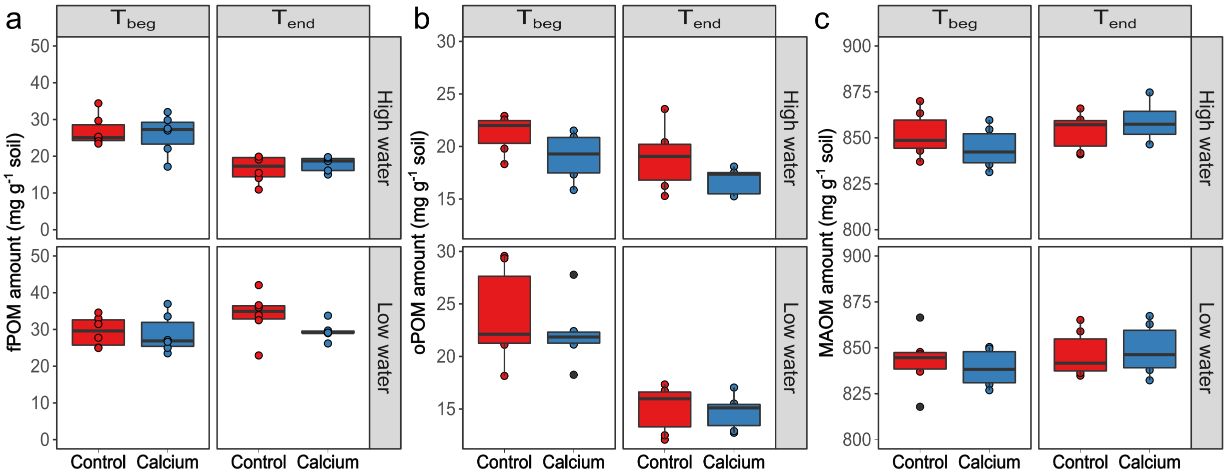

Supplementary Fig. 6. Mass distribution (mg fraction g^{-1} soil) of fPOM (a), oPOM (b), and MAOM (c) fractions in Ca-treated and control soils incubated at low and high water content and sampled at T_{beg} and T_{end}. Boxplots are constructed from n = 6 independent samples. See Supplementary Table 5 for ANOVA model output and P-values derived from pairwise comparison between Control and Calcium.

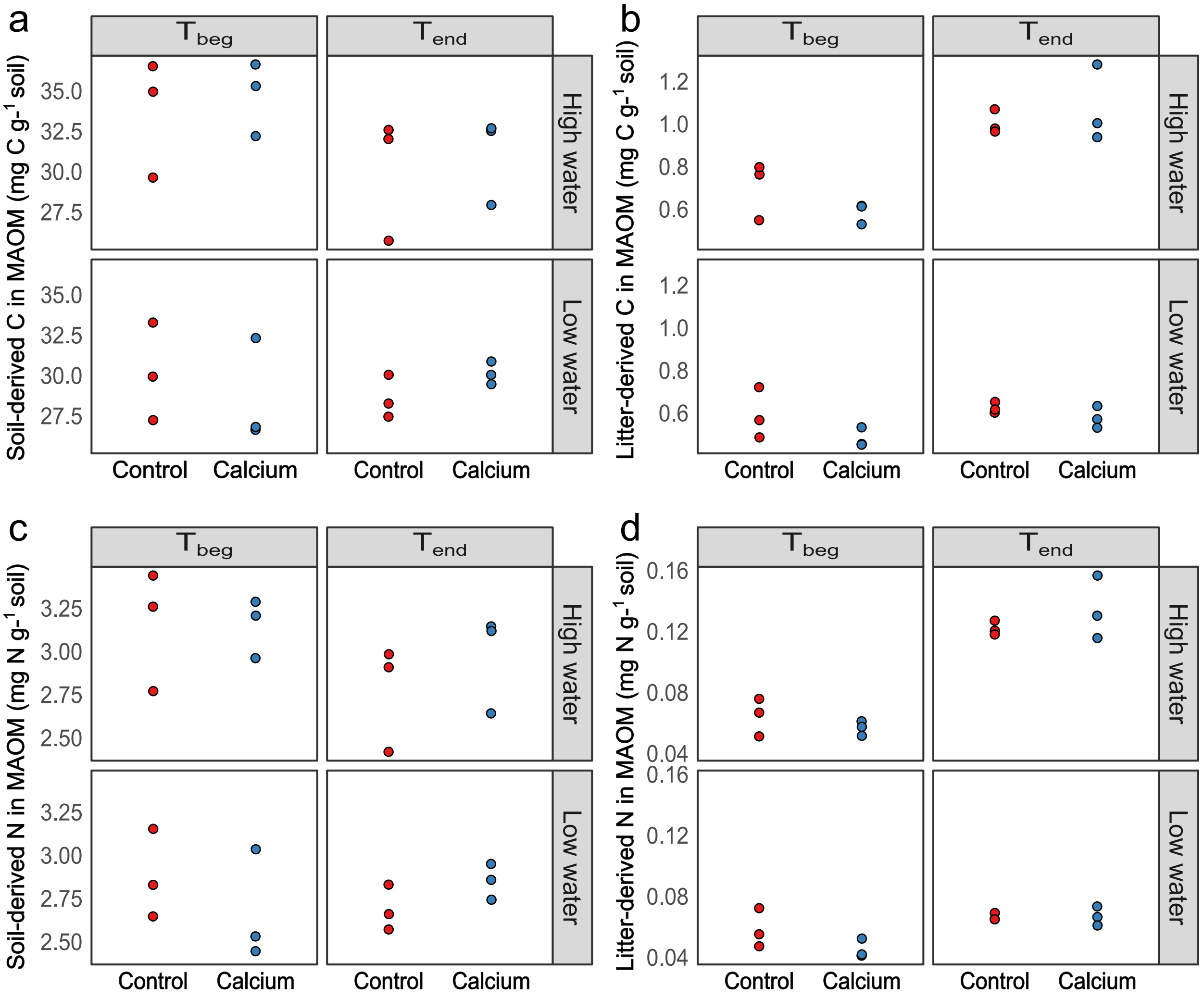

Supplementary Fig. 7. Soil-derived C (a), and N (c), and litter derived C (b), and N (d) in the mineral associated organic matter (MAOM) fraction of Ca-treated and control soils incubated at low and high water content and sampled at T_{beg} and T_{end}. See Supplementary Table 6 for ANOVA model output and and P-values derived from pairwise comparison between Control and Calcium. Statistics were derived from *n* = 3 independent samples.

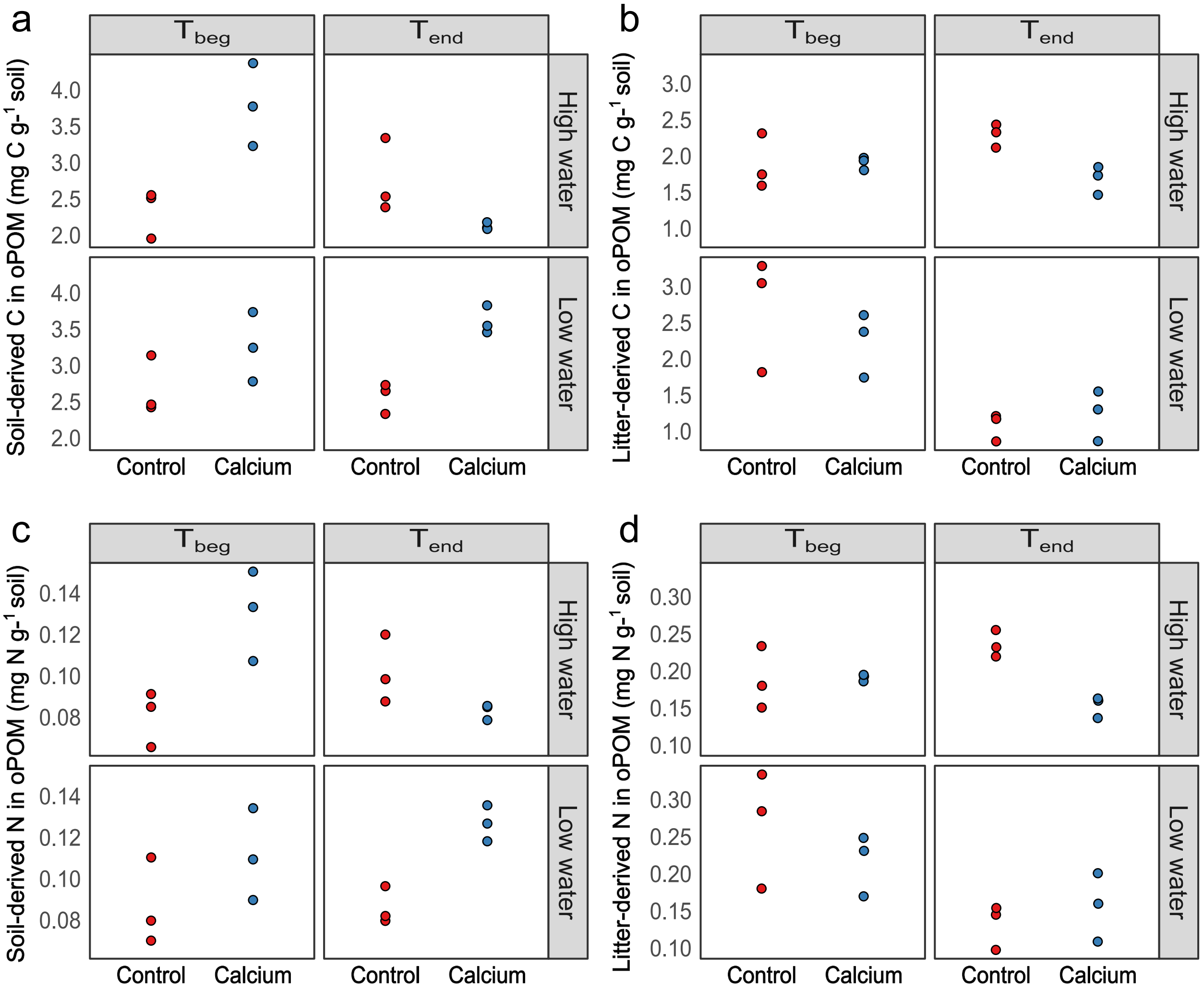

Supplementary Fig. 8. Soil-derived C (a), and N (c), and litter derived C (b), and N (d) in the occluded particulate organic matter (oPOM) fraction of Ca-treated and control soils incubated at low and high water content and sampled at T_{beg} and T_{end}. See Supplementary Table 6 for ANOVA model output and and P-values derived from pairwise comparison between Control and Calcium. Statistics were derived from *n* = 3 independent samples.

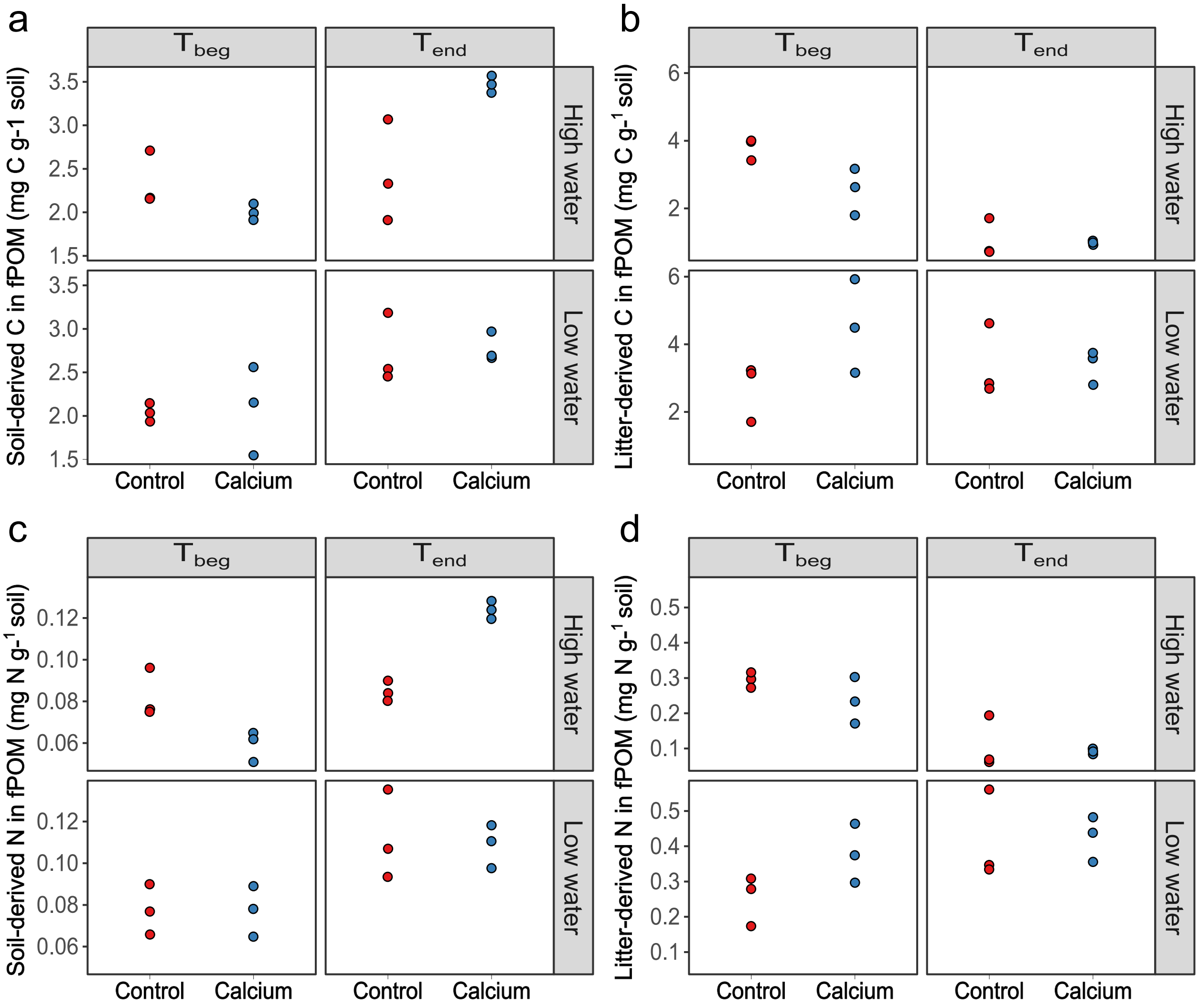

Supplementary Fig. 9. Soil-derived C (a), and N (c), and litter derived C (b), and N (d) in the free particulate organic matter (fPOM) fraction of Ca-treated and control soils incubated at low and high water content and sampled at T_{beg} and T_{end}. See Supplementary Table 6 for ANOVA model output and pairwise comparison between Control and Calcium. Statistics were derived from n = 3 independent samples.

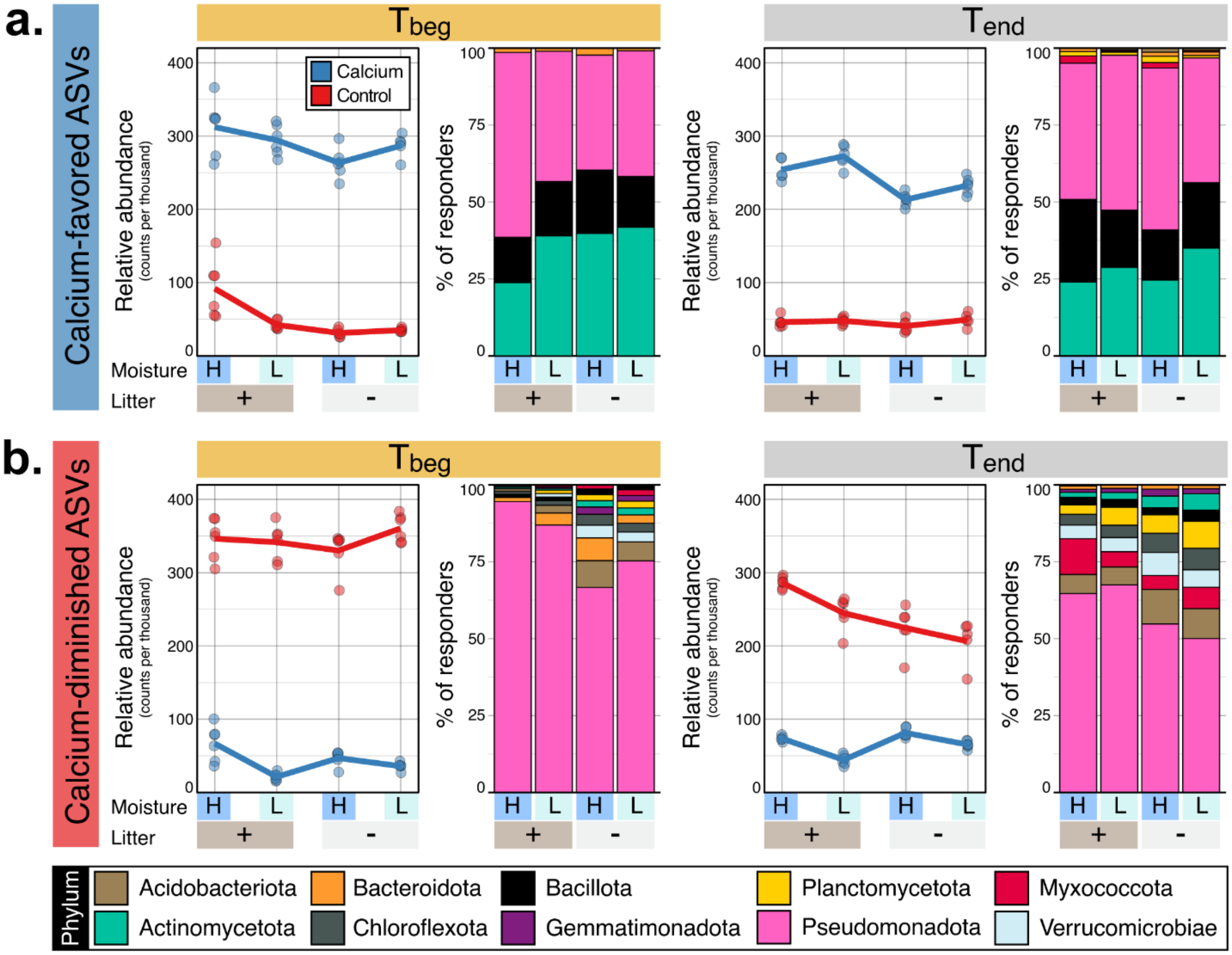

Supplementary Fig. 10. An overview of bacterial populations that were (a) more or (b) less differentially abundant in Ca-treated versus control soils. Each panel contains a plot of the aggregated relative abundance of each population (counts per thousand reads), and an overview of the taxonomic classification of the most abundant groups ($\bar{x} > 0.2$ counts per thousand) at rank Phylum as a % of reads for each population.

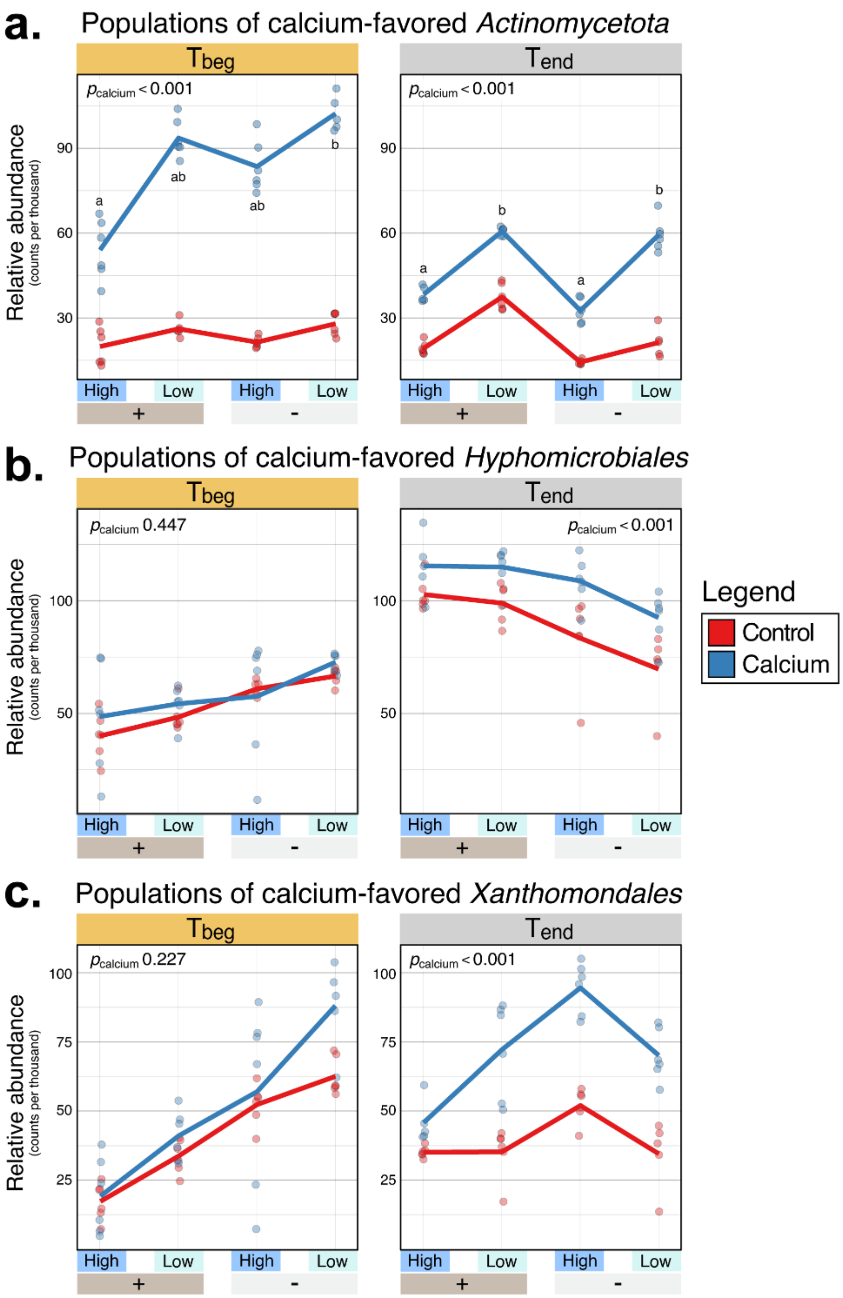

Supplementary Fig. 11. Ca-treatment produced an increase in relative abundance of major bacterial groups relative to control soils, including members of the (a) *Actinomycetota*,(b) *Hyphomicrobiales*¸ and (c) *Xanthomondales*. Notably, time-dependent differences in the effects of Ca were observed, with the increase in *Actinomycetota* most pronounced at T_{beg}, and subsiding at T_{end}, while the enrichment of *Hyphomicrobiales* and *Xanthomondales* by Ca-treatment was only apparent at T_{end}. The plot shows the aggregated relative abundance of several genera for each Phylum or Class. The significance of the main effect of calcium is displayed in each panel, according to results of linear regression models. Lettering denotes significant differences in mean DNA yield among litter and moisture treatment according to TukeyHSD ($p_{adj} < 0.05$). Statistical significance was tested using unpaired, two-sided t-tests on n = 6 independent samples.

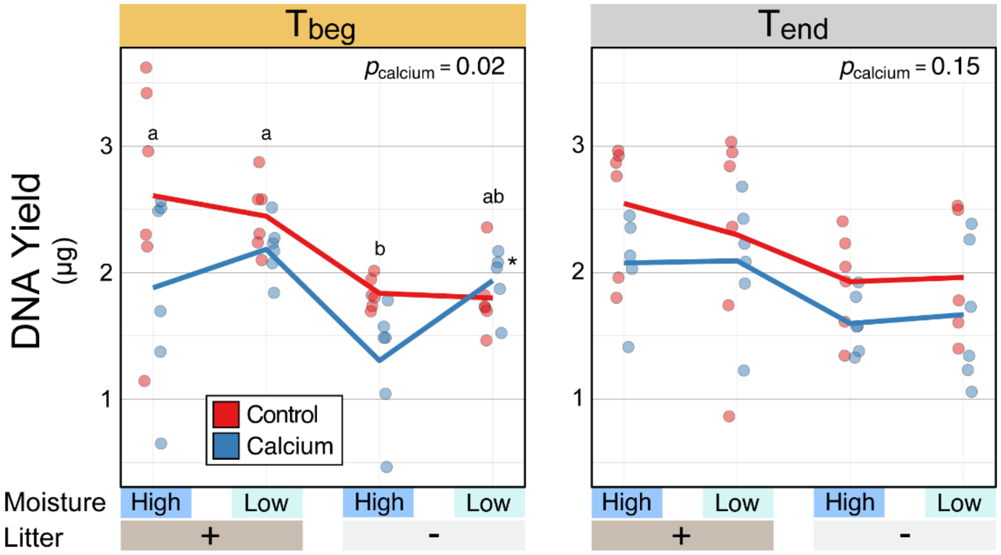

Supplementary Fig. 12. DNA yields from litter and calcium amended soil were significantly higher than controls at the earliest time point. Differences in DNA yield among soil treatments were not significant by the end of the incubation period. The significance of the main effect of calcium is displayed in each panel, according to results of linear regression models. Significant interactions between calcium and treatment (litter + moisture) were denoted by asterisks. Lettering denotes significant differences in mean DNA yield among litter and moisture treatment according to TukeyHSD ($p_{adj} < 0.05$). Statistical significance was tested using unpaired, two-sided t-tests on n = 6 independent samples.

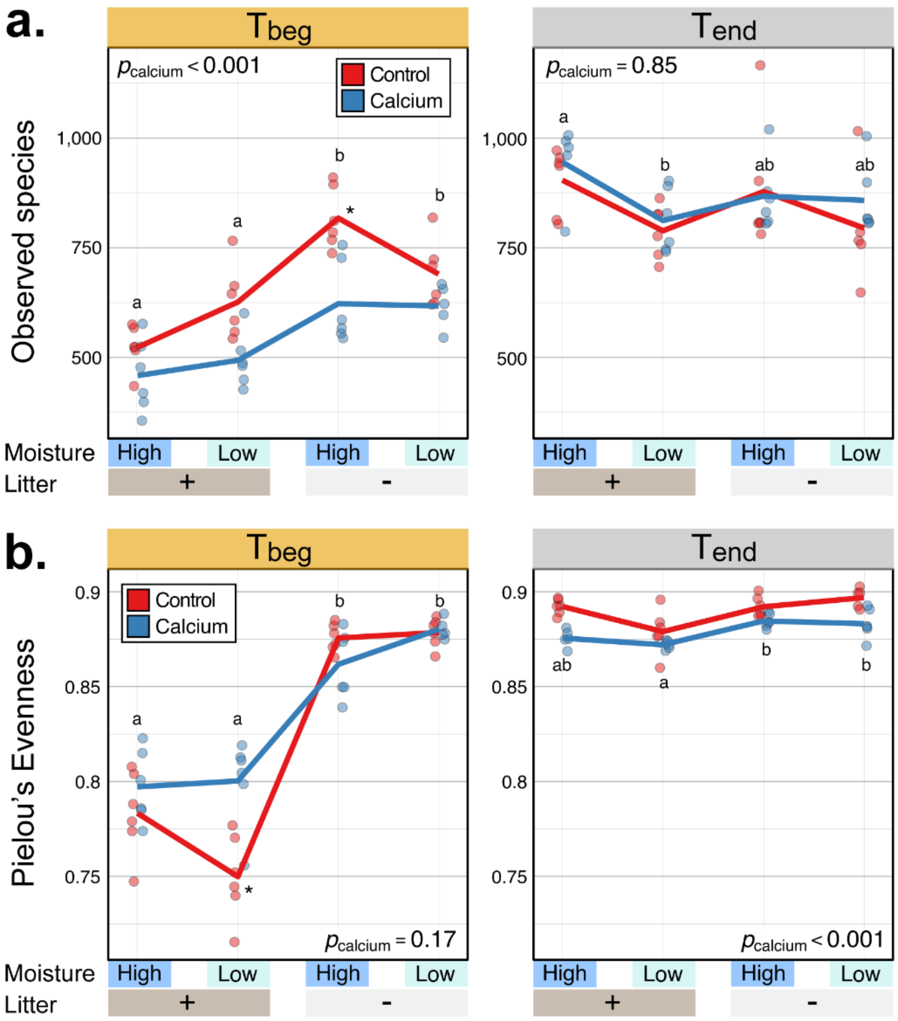

Supplementary Fig. 13. The alpha-diversity of bacterial communities differed among soil treatments according to (a) observed species richness and (b) Pielou's evenness. The effect of treatments varied; however, all communities had increased in richness and evenness over time. The significance of the main effect of calcium is displayed in each panel, according to results of linear regression models. Significant interactions between calcium and treatment (litter + moisture) were denoted by asterisks. Lettering denotes significant differences in means among litter and moisture treatment according to TukeyHSD ($p_{adj} < 0.05$). Statistical significance was tested using unpaired, two-sided t-tests on $n = 6$ independent samples.

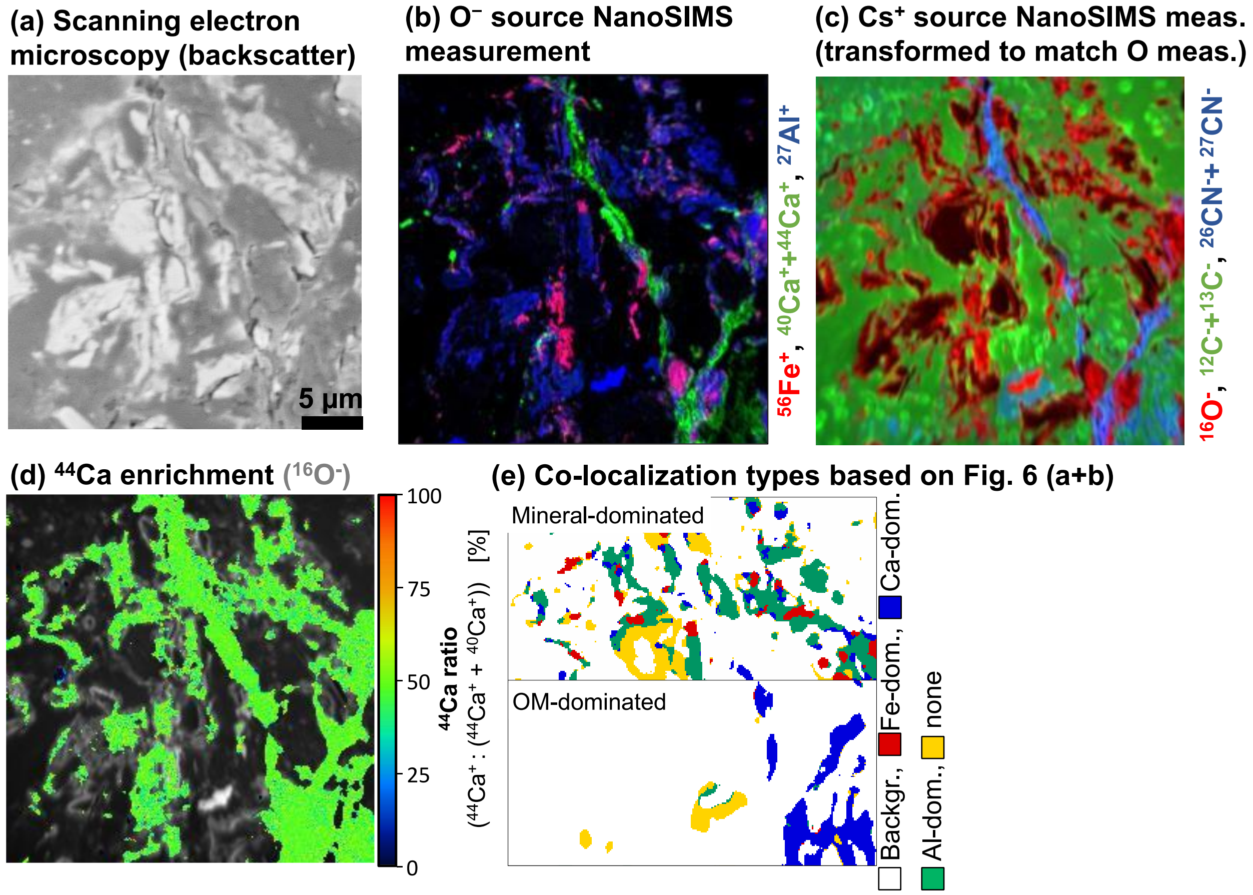

Supplementary Fig. 14. Overview of NanoSIMS analysis. Detection of regions of interest by scanning electron microscopy with backscatter detector (a). Measurement of secondary ion distribution by NanoSIMS using the O^- source (b) and Cs^+ source (c) at the same locations. The Cs^+ source was transformed to optimize the spatial correlation with the O^- source. Spatial distribution of ^{44}Ca enrichment (d). Co-localization types disentangled using machine-learning algorithm-based multi-channel image segmentation (Fig. 6a-b).

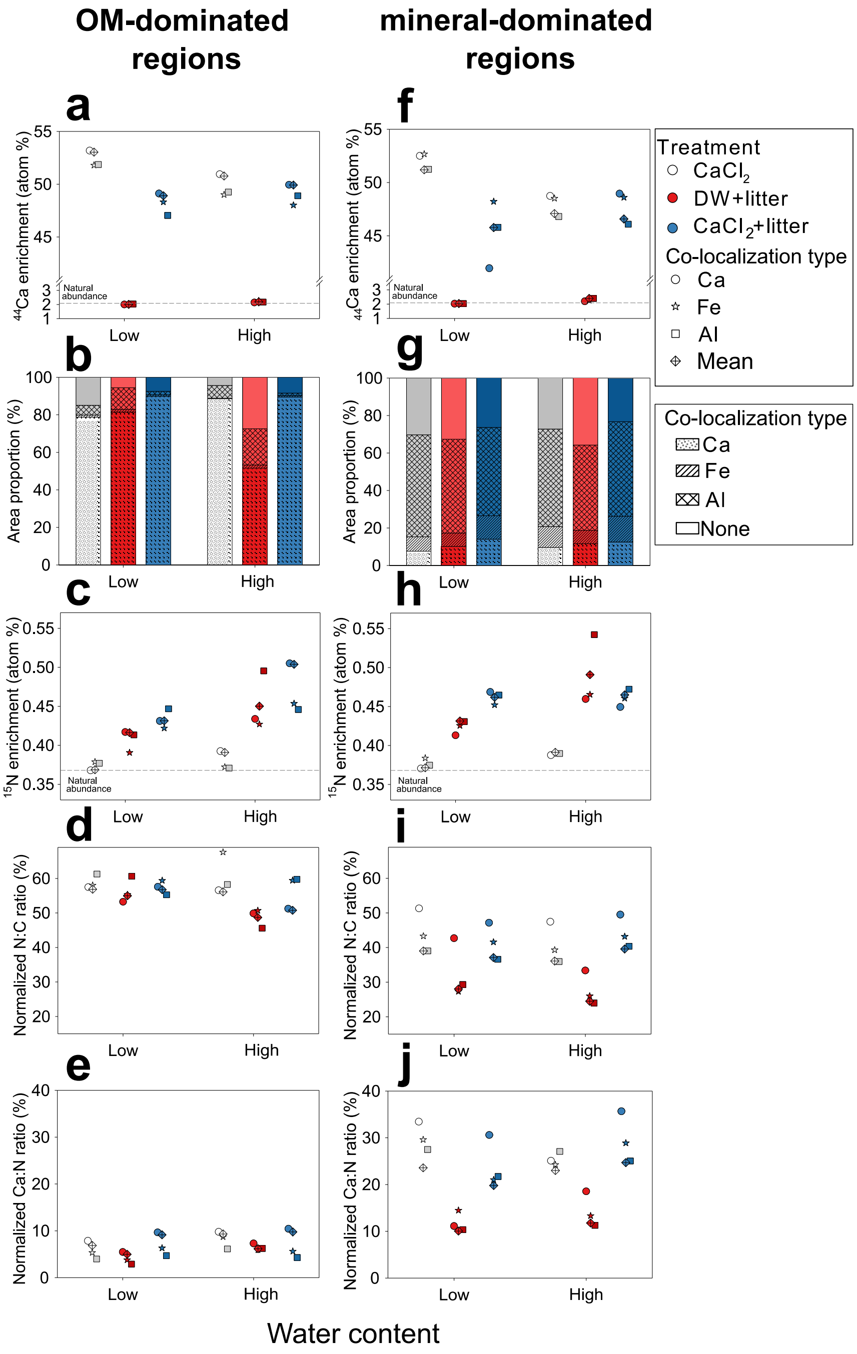

Supplementary Fig. 15. NanoSIMS analysis of soil incubated at low or high water content. ^{44}Ca enrichment ($^{44}Ca^+$):($^{40}Ca^+$ + $^{44}Ca^+$) in Ca, Al, and Fe segments in OM-dominated regions (a) and mineral-dominated regions (f). Area proportion of Ca, Al, and Fe co-localization with OM-dominated regions (b) and mineral-dominated regions (g). Quantification of ^{15}N enrichment ($^{12}C^{15}N^-$:($^{12}C^{14}N^-$ + $^{12}C^{15}N^-$), N:C ratio ($^{12}C^{14}N^-$):($^{12}C^-$ + $^{12}C^{14}N^-$) (E), and Ca:N ratio (($^{40}Ca^+$ + $^{44}Ca^+$):($^{40}Ca^+$ + $^{44}Ca^+$ + $^{12}C^{14}N^-$ + $^{12}C^{15}N^-$)) in Ca, Al, and Fe segments in OM-dominated regions (c-e) and mineral-dominated regions (h-j). All values are based on means derived from *n* = 9 images from control soils and *n* = 12 images from calcium-treated soils.

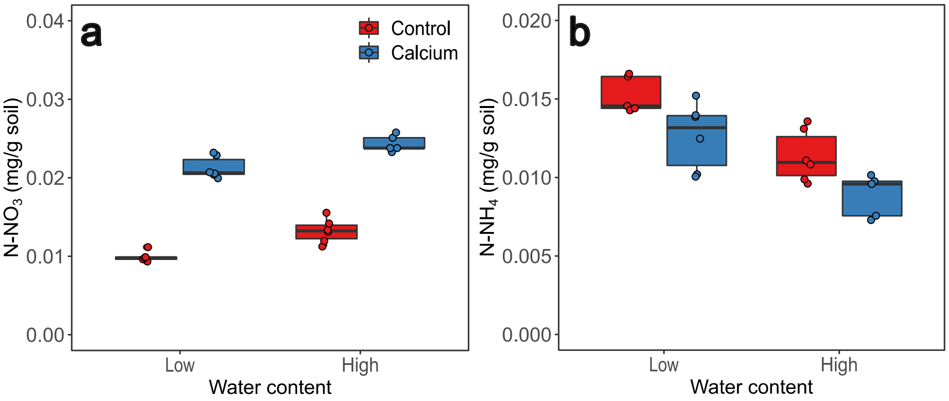

Supplementary Fig. 16. Concentration of nitrate N (a) and ammonium N (b) in calcium-treated and control soils incubated with labeled litter at low or high water content and sampled at T_{end}. Statistical significance was tested using unpaired, two-sided t-tests on data from *n* = 5-6 independent replicates from each treatment combination.

These results show that inorganic N species constituted up to ~1% of total N (ca. 0.34 mg g^{-1} soil) and therefore were of negligible importance to our NanoSIMS results.

Notably, differences between treatments were observed especially for nitrate N (P <0.001) but also for ammonium N (P = 0.0019). No significant interaction between treatment and water content was observed. Higher nitrification in Ca-treated soil and higher water content is consistent with greater microbial activity. Lower ammonium concentration in Ca-treated and high water content soils can be explained by a slower rate of ammonification compared to nitrification. In that case, although ammonification increased, ammonium still decreased.

References

1. Wojdyr, M. Fityk : a general-purpose peak fitting program. *J. Appl. Crystallogr.* **43**, 1126–1128 (2010).
2. Ravel, B. & Newville, M. ATHENA , ARTEMIS , HEPHAESTUS : data analysis for X-ray absorption spectroscopy using IFEFFIT. *J. Synchrotron Radiat.* **12**, 537–541 (2005).
3. Solomon, D. *et al.* Carbon (1s) NEXAFS Spectroscopy of Biogeochemically Relevant Reference Organic Compounds. *Soil Sci. Soc. Am. J.* **73**, 1817 (2009).
4. Politi, Y. *et al.* Transformation mechanism of amorphous calcium carbonate into calcite in the sea urchin larval spicule. *Proc. Natl. Acad. Sci.* **105**, 17362–17366 (2008).
5. Lehmann, J., Kinyangi, J. & Solomon, D. Organic matter stabilization in soil microaggregates: Implications from spatial heterogeneity of organic carbon contents and carbon forms. *Biogeochemistry* **85**, 45–57 (2007).
6. Toplak, M. *et al.* Quasars/orange-spectroscopy: Release 0.5.8. (2021) doi:10.5281/ZENODO.4617978.
7. Herrmann, A. M. *et al.* A novel method for the study of the biophysical interface in soils using nano-scale secondary ion mass spectrometry. *Rapid Commun. Mass Spectrom.* **21**, 29–34 (2007).
8. Gormanns, P., Reckow, S., Poczatek, J. C., Turck, C. W. & Lechene, C. Segmentation of Multi-Isotope Imaging Mass Spectrometry Data for Semi-Automatic Detection of Regions of Interest. *PLoS One* **7**, e30576 (2012).
9. Schindelin, J., Rueden, C. T., Hiner, M. C. & Eliceiri, K. W. The ImageJ ecosystem: An open platform for biomedical image analysis. *Mol. Reprod. Dev.* **82**, 518–529 (2015).
10. Bogovic, J. A., Hanslovsky, P., Wong, A. & Saalfeld, S. Robust registration of calcium images by learned contrast synthesis. in *2016 IEEE 13th International Symposium on Biomedical Imaging (ISBI)* 1123–1126 (2016). doi:10.1109/ISBI.2016.7493463.
11. Berg, S. *et al.* ilastik: interactive machine learning for (bio)image analysis. *Nat. Methods* **16**, 1226–1232 (2019).
12. Schweizer, S. A., Hoeschen, C., Schlüter, S., Kögel-Knabner, I. & Mueller, C. W. Rapid soil formation after glacial retreat shaped by spatial patterns of organic matter accrual in microaggregates. *Glob. Chang. Biol.* **24**, 1637–1650 (2018).
13. Kozich, J. J., Westcott, S. L., Baxter, N. T., Highlander, S. K. & Schloss, P. D. Development of a dual-index sequencing strategy and curation pipeline for analyzing amplicon sequence data on the Miseq Illumina sequencing platform. *Appl. Environ. Microbiol.* **79**, 5112–5120 (2013).
14. Zwetsloot, M. J. *et al.* Prevalent root-derived phenolics drive shifts in microbial community composition and prime decomposition in forest soil. *Soil Biol. Biochem.* **145**, 107797 (2020).

15. Wilhelm, R. C. Following the terrestrial tracks of *Caulobacter* - redefining the ecology of a reputed aquatic oligotroph. *ISME J.* **12**, 3025–3037 (2018).

16. McBride, M. J. Bacterial gliding motility: multiple mechanisms for cell movement over surfaces. *Annu. Rev. Microbiol.* **55**, 49–75 (2001).

17. Wolf, A. B., Vos, M., De Boer, W. & Kowalchuk, G. A. Impact of matric potential and pore size distribution on growth dynamics of filamentous and non-filamentous soil bacteria. *PLoS One* **8**, e83661 (2013).

18. Wilhelm, R. C., Pepe-Ranney, C., Weisenhorn, P., Lipton, M. & Buckley, D. H. Competitive exclusion and metabolic dependency among microorganisms structure the cellulose economy of an agricultural soil. *MBio* **12**, 1–19 (2021).

19. Womack, B. J., Gilmore, D. F. & White, D. Calcium requirement for gliding motility in myxobacteria. *J. Bacteriol.* **171**, 6093–6096 (1989).

20. Garcia, R. & Müller, R. The Family Haliangiaceae. in *The Prokaryotes: Deltaproteobacteria and Epsilonproteobacteria* vol. 9783642390 1–413 (2014).

21. Mitsumori, M., Xu, L. M., Kajikawa, H. & Kurihara, M. Properties of cellulose-binding modules in endoglucanase F from Fibrobacter succinogenes S85 by means of surface plasmon resonance. *FEMS Microbiol. Lett.* **214**, 277–281 (2002).

22. Stehr, K. R. *et al.* Effect of replacing barley silage with calcium oxide-treated barley straw on rumen fermentation, rumen microbiota, nutrient digestibility, and growth performance of finishing beef cattle. *Can. J. Anim. Sci.* **101**, 493–506 (2021).

23. Marsden, A. E., Grudzinski, K., Ondrey, J. M., DeLoney-Marino, C. R. & Visick, K. L. Impact of salt and nutrient content on biofilm formation by Vibrio fischeri. *PLoS One* **12**, 1–19 (2017).

24. Wang, T., Flint, S. & Palmer, J. Magnesium and calcium ions: roles in bacterial cell attachment and biofilm structure maturation. *Biofouling* **35**, 959–974 (2019).

25. Havenner, J. A., McCardell, B. A. & Weiner, R. M. Development of defined, minimal, and complete media for the growth of *Hyphomicrobium neptunium*. *Appl. Environ. Microbiol.* **38**, 18–23 (1979).

26. Volk, E., Iden, S. C., Furman, A., Durner, W. & Rosenzweig, R. Biofilm effect on soil hydraulic properties: Experimental investigation using soil-grown real biofilm. *Water Resour. Res.* **52**, 5813–5828 (2016).

27. Butcher, K. R., Nasto, M. K., Norton, J. M. & Stark, J. M. Physical mechanisms for soil moisture effects on microbial carbon-use efficiency in a sandy loam soil in the western United States. *Soil Biol. Biochem.* **150**, 107969 (2020).

28. Shabtai, I. A., Shenker, M., Edeto, W. L., Warburg, A. & Ben-Hur, M. Effects of land use on structure and hydraulic properties of Vertisols containing a sodic horizon in northern Ethiopia. *Soil Tillage Res.* **136**, (2014).
