# Supplementary material for: Calcium promotes persistent soil organic matter by altering microbial transformation of plant litter
Source: Nat Commun. 2023 Oct 19;14:6609. doi: 10.1038/s41467-023-42291-6 (PMC10587086; doi:10.1038/s41467-023-42291-6)
Supplement: Supplementary file 3 — Description of Additional Supplementary Files [file 41467_2023_42291_MOESM3_ESM.pdf]

Description of Additional Supplementary Files:

Supplementary Data 1: A complete list of ASVs indicative of calcium and litter amendment, or soil moisture content. All responding ASVs had a minimum 3-fold increase in relative abundance in soils. For example, an indicator of 'calcium' was significantly differentially more abundant in calcium-amended soils. The average and maximum relative abundance of each ASV was based on all samples. A representative 16S rRNA gene sequence has been provided for each. Please note that this table is constructed with taxonomic classification from Silva X, which do not reflect the most current nomenclature recommended by the National Center for Biotechnology Information (NCBI). The names in this table can be searched using the NCBI Taxonomy Browser to obtain the corresponding current nomenclature.
