# Supplementary material for: Calcium promotes persistent soil organic matter by altering microbial transformation of plant litter
Source: Nat Commun. 2023 Oct 19;14:6609. doi: 10.1038/s41467-023-42291-6 (PMC10587086; doi:10.1038/s41467-023-42291-6)
Supplement: Supplementary file 5 — Reporting Summary [file 41467_2023_42291_MOESM5_ESM.pdf]

nature portfolio

Corresponding author(s): Itamar Shabtai

Last updated by author(s): Sep 18, 2023

Reporting Summary

Nature Portfolio wishes to improve the reproducibility of the work that we publish. This form provides structure for consistency and transparency in reporting. For further information on Nature Portfolio policies, see our Editorial Policies and the Editorial Policy Checklist.

Statistics

For all statistical analyses, confirm that the following items are present in the figure legend, table legend, main text, or Methods section.

n/a	Confirmed	
☐	☒	The exact sample size (*n*) for each experimental group/condition, given as a discrete number and unit of measurement
☐	☒	A statement on whether measurements were taken from distinct samples or whether the same sample was measured repeatedly
☐	☒	The statistical test(s) used AND whether they are one- or two-sided<br>*Only common tests should be described solely by name; describe more complex techniques in the Methods section.*
☒	☐	A description of all covariates tested
☐	☒	A description of any assumptions or corrections, such as tests of normality and adjustment for multiple comparisons
☐	☒	A full description of the statistical parameters including central tendency (e.g. means) or other basic estimates (e.g. regression coefficient) AND variation (e.g. standard deviation) or associated estimates of uncertainty (e.g. confidence intervals)
☐	☒	For null hypothesis testing, the test statistic (e.g. *F*, *t*, *r*) with confidence intervals, effect sizes, degrees of freedom and *P* value noted<br>*Give P values as exact values whenever suitable.*
☒	☐	For Bayesian analysis, information on the choice of priors and Markov chain Monte Carlo settings
☒	☐	For hierarchical and complex designs, identification of the appropriate level for tests and full reporting of outcomes
☐	☒	Estimates of effect sizes (e.g. Cohen's *d*, Pearson's *r*), indicating how they were calculated

Our web collection on statistics for biologists contains articles on many of the points above.

Software and code

Policy information about availability of computer code

Data collection	No software was used to collect data.
Data analysis	All statistical analyses were out in R 4.2.0 in the RStudio IDE 2023.06.0+421, using the tidyverse, emmeans, vegan, DESeq2, Rtsne, and phyloseq packages. The ImageJ software (Fiji/ImageJ 1.51h; https://imagej.nih.gov/ij) with the OpenMIMS plugin was used to compute ratios for distinct regions of interests and apply dead time and drift corrections in the NanoSIMS measurements, and the BigWarp plugin was used to match Cs and O data. Quasar 1.5.0 was used to preprocess the FTIR microscopy data and construct spectral maps. Athena 0.9.26 was used to edge-step normalize the NEXAFS data and Fityk 1.3.1 was used to deconvolute the NEXAFS spectra.

For manuscripts utilizing custom algorithms or software that are central to the research but not yet described in published literature, software must be made available to editors and reviewers. We strongly encourage code deposition in a community repository (e.g. GitHub). See the Nature Portfolio guidelines for submitting code & software for further information.

Data

Policy information about availability of data

All manuscripts must include a data availability statement. This statement should provide the following information, where applicable:

- Accession codes, unique identifiers, or web links for publicly available datasets
- A description of any restrictions on data availability
- For clinical datasets or third party data, please ensure that the statement adheres to our policy

All data required to reproduce the manuscript results are available at Zenodo: 10.5281/zenodo.8139855. A complete list of amplicon sequence variants ASVs) is

provided in the Supplementary Data. All raw sequencing data and associated metadata was archived under the NCBI BioProject accession: PRJEB48763 available at https://www.ncbi.nlm.nih.gov/bioproject/?term=PRJEB48763.

Research involving human participants, their data, or biological material

Policy information about studies with human participants or human data. See also policy information about sex, gender (identity/presentation), and sexual orientation and race, ethnicity and racism.

Reporting on sex and gender	N/A
Reporting on race, ethnicity, or other socially relevant groupings	N/A
Population characteristics	N/A
Recruitment	N/A
Ethics oversight	N/A

Note that full information on the approval of the study protocol must also be provided in the manuscript.

Field-specific reporting

Please select the one below that is the best fit for your research. If you are not sure, read the appropriate sections before making your selection.

☐ Life sciences ☐ Behavioural & social sciences ☒ Ecological, evolutionary & environmental sciences

For a reference copy of the document with all sections, see nature.com/documents/nr-reporting-summary-flat.pdf

Ecological, evolutionary & environmental sciences study design

All studies must disclose on these points even when the disclosure is negative.

Study description	We investigated the role of calcium on microbial processing of litter and cycling of litter-derived carbon and nitrogen. The study included a ~120-130 day incubation in which we studied how calcium influenced the mineralization and transformation of native soil organic matter and added isotopically labeled litter, the formation of organo-mineral interactions, and the microbial community composition.<br>The incubation was set up in a full factorial design, with six replicated experimental units (microcosms) for each treatment factor combination (calcium addition, litter addition, soil water content). Each factor had two levels: calcium addition or control, with or without 13C15N labeled litter, and low and high water content. During incubation, respiration (CO2) measurements were carried out randomly on three of the six replicates. The experiment was duplicated to allow destructive sampling after four days (referred to as Tbeg in the manuscript) and at the end of the incubation (Tend), adding a time variable. Some of the analyses performed following destructive sampling were done on all six replicates and some were done on triplicates. Triplicated samples were made by randomly pairing up the six replicates into three pairs and combining the pairs.
Research sample	The soil used in this study was sampled from a fallow plot within an experimental field (see additional details under "Locations"). The litter material used in this study was from a willow (Salix viminalis x S.miyabeana) which is common in the area the soil was collected from. The soil, used in this study, an Alfisol, was chosen to represent a common agricultural soil. Alfisols are widely distributed in many parts of the Northeast and Midwest US and are widely used for agriculture.
Sampling strategy	To account for field variation, we collected soil from 4 corners of approximately a 4x4 m square. No pre-calculation of samples size were performed, however, several previous studies conducted on this field site have used this sampling scheme to satisfactorily characterize subplots within this field. Only the Ap horizon was sampled. In the lab, root material and rocks were removed and the soil material was homogenized by sieving through a 2-mm sieve. This was done to reduce variability between experimental units (microcosms). The leaf litter was finely ground and added individually to each soil microcosm to obtain a uniform litter/soil ratio. The incubation was carried out on six replicates per treatment.
Data collection	Itamar Shabtai carried out and performed the soil respiration measurements using KOH alkali traps, the microbial biomass fumigation extraction and the soil density fractionation. Organic carbon in solution samples were analyzed by Itamar Shabtai (Shimadzu TOC-VCPN), and the solid samples were analyzed on a Delta V, Thermo Scientific, Germany) coupled to an elemental analyzer (NC2500, Carlo Erba, Italy). SEM and NanoSIMS imaging and analyses was done by Carmen Hoschen and Steffen Schweizer. FTIR microscopy and NEXAFS was carried out by Itamar Shabtai at the Canadian Light Source synchrotron. DNA extraction and 16S rRNA sequencing was done by Roland Wilhelm. DNA was extracted using the Qiagen PowerSoil kits, according to the manufacturer's protocol, and PCR was used to amplify the 16S rRNA gene (V4 region; 515f/806r). Amplicon sequencing data was generated using Illumina MiSeq and the raw sequencing data was archived at the NCBI (BioProject accession PRJEB48763) with metadata reported according to the GSC MIxS Soil environmental checklist.
Timing and spatial scale	The incubation experiment took place between 04/15/2020-08/25/2020. Respiration measurements were done on days 1, 2, 3, 4, 6, 8, 11, 15, 21, 30, 44, 61, 91. Respiration measurement was also taken on 112 of the incubation (for both water contents) and day 135 only for the low water content microcosms. The decreasing frequency of respiration measurements captures the typical CO2 release curve of incubated soil which decreases with time. The incubation length was set to allow approximately 50% (in actuality between

	41 and 47.5%) of the added litter to be decomposed. We deemed this to be a suitable threshold that yields substantial amounts of processed labeled carbon and nitrogen while maintaining a substrate source for microbes. Microbial biomass extraction was performed in October 2020 and density fractionation was performed in November 2020, each lasted approximately one week. FTIR microscopy and NEXAFS spectroscopy was done at the Canadian Light Source synchrotron in January 2021 and August 2021, respectively, and lasted approximately 3 days each. DNA extraction and sequencing was done in September 2021 and took approximately 10 days. NanoSIMS was done in April 2022 and lasted for two weeks. Bulk analytical data is taken from cm scale; FTIR data is taken from the micron scale, and NanoSIMS is taken form the sub-micron scale.
Data exclusions	We experienced a technical failure during the analysis of dissolved total nitrogen in microbial biomass extraction samples. Therefore, we excluded all nitrogen data from the microbial biomass results. During density fractionation one sample was lost due to spillage. Additionally, all FTIR spectra with absorbance < 0.4 at 1035 cm-1 wavenumber was discarded. This was done to remove spectra with low signal/noise ratio. No other sample was excluded.
Reproducibility	Preliminary experiments were performed to determine the appropriate amount of soil, volume of CO2 alkali trap (KOH), and KOH concentration in each microcosm to achieve detectable and repeatable amounts of CO2 in the sampling timing planned (see Timing and Spatial scale). We found that for ~9 g of soil (the amount required for destructive sampling and analyses) in a 473 mL Mason jar, 20 mL of 0.18M KOH was required. We also performed a preliminary fractionation experiment to compare mass and fraction recovery using ultrasonication vs shaking glass beads to disrupt aggregates prior to oPOM and MAOM collection, and found no differences between methods and therefore decided to use glass beads which enables higher throughput. The experimental results show typical reproducibility for the types of soil analyses done here. Both the preliminary incubation and fractionation experiment were performed once before the main experiment.
Randomization	Microcosms location and order of measurement was fully randomized.
Blinding	Microcosms were given unique ID numbers (1, 2, 3, ...etc.) and all containers and vials were labeled using unique ID numbers. During sample processing blinding was not possible because some replicates from the same treatments were grouped, thus group allocation had to be known. During data analyses, raw data was combined with sample metadata which contained the treatment factor levels of each microcosm. However, the data analysis scripts were applied to all samples simultaneously, thus investigators were effectively blind to group allocations during data analysis.

Did the study involve field work? ☒ Yes ☐ No

Field work, collection and transport

Field conditions	Soils were collected on March 3, 2020 (10 °C, no precipitation). The incubation experiment was carried out in a temperature controlled environment at 20 °C.
Location	A Mollic Endoaqualf silt loam was collected at 0 – 15 cm from a fallow plot located in an experimental bioenergy feedstock field in Ithaca, NY (42º N 28.20', 76º W 25.94'). The field has not been plowed since at least 1954, and since then has been under perennial grass and broadleaf forbs cover and has been mowed every several years. The field has been completely fallow since 2005 and has received no fertilizer. The mean annual temperature and precipitation at the site were 10 °C and 940 mm, respectively.
Access & import/export	All samples were collected with permission from the site manager. No permits were required.
Disturbance	No significant disturbance was caused by the collection of the soil in the field.

Reporting for specific materials, systems and methods

We require information from authors about some types of materials, experimental systems and methods used in many studies. Here, indicate whether each material, system or method listed is relevant to your study. If you are not sure if a list item applies to your research, read the appropriate section before selecting a response.

Materials & experimental systems

n/a	Involved in the study	
☒	☐	Antibodies
☒	☐	Eukaryotic cell lines
☒	☐	Palaeontology and archaeology
☒	☐	Animals and other organisms
☒	☐	Clinical data
☒	☐	Dual use research of concern
☒	☐	Plants

Methods

n/a	Involved in the study	
☒	☐	ChIP-seq
☒	☐	Flow cytometry
☒	☐	MRI-based neuroimaging
